# Directional Connectivity between Frontal and Posterior Brain Regions Is Altered with Increasing Concentrations of Propofol

**DOI:** 10.1371/journal.pone.0113616

**Published:** 2014-11-24

**Authors:** Anu Maksimow, Minna Silfverhuth, Jaakko Långsjö, Kimmo Kaskinoro, Stefanos Georgiadis, Satu Jääskeläinen, Harry Scheinin

**Affiliations:** 1 Department of Perioperative Services, Intensive Care and Pain Medicine, Turku University Hospital, Turku, Finland; 2 Turku PET Centre, University of Turku, Turku, Finland; 3 Department of Computer Science and Engineering, University of Oulu, Oulu, Finland; 4 Intensive Care Unit, Tampere University Hospital, Tampere, Finland; 5 Department of Applied Physics, University of Eastern Finland, Kuopio, Finland; 6 Department of Clinical Neurophysiology, Turku University Hospital, Turku, Finland; 7 Department of Pharmacology, Drug Development and Therapeutics, University of Turku, Turku, Finland; Leibniz Institute for Neurobiology, Germany

## Abstract

Recent studies using electroencephalography (EEG) suggest that alteration of coherent activity between the anterior and posterior brain regions might be used as a neurophysiologic correlate of anesthetic-induced unconsciousness. One way to assess causal relationships between brain regions is given by renormalized partial directed coherence (rPDC). Importantly, directional connectivity is evaluated in the frequency domain by taking into account the whole multichannel EEG, as opposed to time domain or two channel approaches. rPDC was applied here in order to investigate propofol induced changes in causal connectivity between four states of consciousness: awake (AWA), deep sedation (SED), loss (LOC) and return of consciousness (ROC) by gathering full 10/20 system human EEG data in ten healthy male subjects. The target-controlled drug infusion was started at low rate with subsequent gradual stepwise increases at 10 min intervals in order to carefully approach LOC (defined as loss of motor responsiveness to a verbal stimulus). The direction of the causal EEG-network connections clearly changed from AWA to SED and LOC. Propofol induced a decrease (p = 0.002–0.004) in occipital-to-frontal rPDC of 8-16 Hz EEG activity and an increase (p = 0.001–0.040) in frontal-to-occipital rPDC of 10–20 Hz activity on both sides of the brain during SED and LOC. In addition, frontal-to-parietal rPDC within 1–12 Hz increased in the left hemisphere at LOC compared to AWA (p = 0.003). However, no significant changes were detected between the SED and the LOC states. The observed decrease in back-to-front EEG connectivity appears compatible with impaired information flow from the posterior sensory and association cortices to the executive prefrontal areas, possibly related to decreased ability to perceive the surrounding world during sedation. The observed increase in the opposite (front-to-back) connectivity suggests a propofol concentration dependent association and is not directly related to the level of consciousness *per se*.

## Introduction

The focus of recent research aiming to solve the mechanisms of anesthetic-induced loss of consciousness (LOC) has moved from the analysis of local findings towards the evaluation of alterations in functional integration of the neural networks in the brain [1], [2], [3], [4], [5]. Anesthetic-induced changes in connectivity between remote brain structures can be explored using, e.g., functional magnetic resonance imaging (fMRI) [4], [6], [7], positron emission tomography (PET) [1], [8] and electroencephalography (EEG) based methods [9], [10], [11]. Studies utilizing these different tools demonstrate impaired thalamocortical [1] and cortico-cortical connectivity [4], [5], [8] during anesthetic-induced unconsciousness as well as during physiological sleep in human subjects [12]. Many of these studies conclude that frontoparietal functional connectivity plays an important role in conscious perception [8], [9], [13].

Several methods capable of detecting causal connectivity can be applied on EEG signals. Among the most promising are multivariate approaches including partial directed coherence (PDC) [14], [15]. Based on the Granger causality principle of forecasting, a priori hypothesis of directional relationships is not required in PDC analysis. In addition, a clear advantage of PDC is its ability to detect direct relations instead of secondary or indirect connections [15], [16]. An improved version, renormalized PDC (rPDC), was developed to overcome some drawbacks of the original PDC approach [17], and was applied in the present study. Both PDC and rPDC are based on multivariate autoregressive modeling of vector processes and explicitly evaluate causal relationships in the frequency domain by taking into account all the available EEG channels. rPDC values directly reflect the strength of the directional influence, while the original PDC measures the strength in relation to the signal source power. Furthermore, in the evaluation of statistically significant connections, rPDC utilizes constant significance levels obtained from a chi square distribution [17]. Therefore, in contrary to PDC, rPDC allows conclusions on the absolute strength of coupling and is suited for comparing the strength at different frequencies or between different pairs of variables.

The aim of this study was to investigate directional connectivity changes between different brain areas in healthy human subjects during slow progression to unconsciousness using single anesthetic agent propofol. To our knowledge, the applicability of PDC for anesthesia research has been previously tested in only one experimental study where cortico-hippocampal functional connectivity in rat brain was assessed during isoflurane anesthesia with promising results [18]. Our approach with rPDC, a novel causal connectivity measure in human anesthesia research, can also be seen as an extension of the recent work applying Granger causality method [19].

## Methods

### Subjects

The study protocol was approved by the Ethics Committee of the Hospital District of Southwest Finland (Turku, Finland) and the Finnish Medicines Agency. After giving written informed consent, 10 healthy (American Society of Anesthesiologists physical status I), right-handed, non-smoking normal weight male subjects aged 19–28 years were enrolled in this open, non-randomized study. None of the subjects had a history of psychiatric disorder, somatic illness, substance abuse, drug allergies, or ongoing medications. Exclusion criteria included susceptibility for nausea, cardiac arrhythmia and hearing impairment. All subjects underwent a detailed pre-study examination comprising of an interview, physical status, laboratory testing including psychometric drug screen and a 12-lead ECG. Subjects refrained from using alcohol or any medication for 48 hours before the study and fasted overnight. No pre-medication was given prior to propofol administration.

### Study design and data acquisition

Data for the present experiment was collected during the dose-finding part of our positron emission tomography study aiming to uncover the neural mechanisms of consciousness [8].

Propofol (Propofol Lipuro 10 mg ml^−1^, B. Braun Melsungen AG, Pfieffewiesen, D-34212 Melsungen, Germany) was administrated intravenously using target controlled infusion (TCI) scheme aiming at pseudo steady-state plasma concentrations escalating at 10 min intervals. A Harvard 22 syringe pump (Harvard Apparatus, South Natick, MA) connected to portable computer and running Stanpump software (by Steven L. Schafer, MD, available at http://www.opentci.org/doku.php?id=code:code) with the Marsh pharmacokinetic model [20] was used. Infusion was started at plasma target concentration of 1.0 µg ml^−1^, followed first by 0.5 µg ml^−1^ target concentration increase and 0.25 µg ml^−1^ increases thereafter (i.e. 1.0–1.5–1.75–2.0–2.25- etc. µg ml^−1^) until LOC was achieved. Throughout the study, consciousness was tested at 5 min intervals (i.e. at 4 and 9 min of each 10 min concentration level) by requesting the subjects to open their eyes. LOC was defined as loss of visible motor response to the request and the sedation state (SED) as the last testing condition before LOC when the subjects could still open their eyes (i.e. 5 min before confirmed LOC). After LOC, the administration of propofol was terminated followed by consciousness-testing at 1 min intervals until return of consciousness (ROC) was detected. ROC was defined as the first eye opening to the request after the discontinuation of propofol infusion.

EEG was continuously recorded using a Galileo (Medtronic, Italy) electroencephalogram acquisition system. Baseline EEG was recorded while the subjects were resting eyes closed before drug administration. The recordings were made with standard International 10/20 Electrode Placement System locations (linked mastoid reference) using ElectroCap with Ag-AgCl electrodes. Electrode impedances were kept below 2 kΩ. The sampling rate was 256 Hz and the signals were band-pass filtered with 0.1–70 Hz frequency range. Recordings began 5 minutes before starting the propofol administration and ended about 10 minutes after ROC, and their total duration varied among subjects. Monitoring also included non-invasive blood pressure (only at the start and end of study), pulse-oximetry, a 3-lead electrocardiogram, and inspiratory and expiratory concentrations of gases (O2, EtCO2). GE Datex-Ohmeda S/5 Anaesthesia Monitor and a portable computer running the S/5 Collect software (Datex-Ohmeda S/5 Collect Version 4.0, GE Healthcare, Helsinki, Finland) were used for monitoring and recording all vital sign values.

### Causality analysis

In the following, Granger-causality for multivariate time series and renormalized partial directed coherence (rPDC) are briefly presented.

A mathematical definition of causality was given by Granger [21]. Granger causality is based on the principle that an observed signal 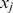 causes another signal 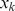, if the additional knowledge of the past of 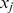 significantly improves prediction of 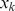. However, the principle holds only if there are no other signals influencing the process [22], [23]. A more general definition handling the whole multivariate structure of a set of measurement channels can be obtained through the theory of vector autoregressive processes. Based on this, directed transfer function (DTF) was introduced in [24] and partial directed coherence (PDC) in [15]. Both approaches allow the investigation of causal relationships in the frequency domain. PDC was recently extended to allow conclusions about the strength of interaction through renormalization [17], see also [25].

For *M* simultaneously observed stationary time series, i.e. 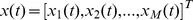, where 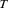 denotes transposition and 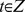, a vector autoregressive model process of order 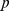, abbreviated VAR[*p*], is given by



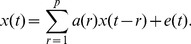
(1)


The elements 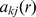 of the matrices 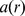, 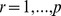, describe the linear relationship between time series, 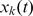 and 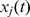 at different time lags 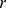, and the vector process 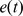 is usually assumed to follow a zero mean multivariate Gaussian white noise process with covariance matrix 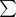. Model properties, stability and stationarity conditions, as well as computational aspects of VAR[*p*] modeling can be found, for instance, in [26]. A process 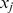 is said to Granger-cause another process 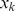 with respect to the full process 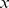 in (1) if the elements 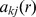, 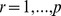, are not all zero or, if linear prediction of 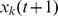 based on the past and present values of all variables but 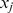 can be improved by adding the past and present values of 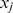. The spectral matrix of the process can be written in the form



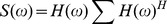
(2)where now 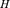 denotes Hermitian transpose. The transfer matrix 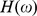 is given by



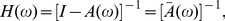
(3)where 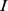 is the identity matrix and 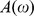 is obtained from the Fourier transform of the coefficients



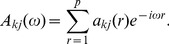
(4)


In [17] the following two-dimensional vector was defined



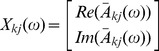
(5)with 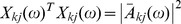. It was further shown that based on the estimated parameters 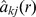 in (4) the corresponding estimator 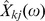 is asymptotically normally distributed with mean 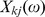 and covariance matrix 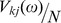, where 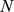 is the number of data points and



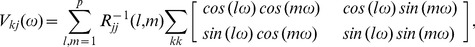
(6)where 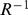 is the inverse of the covariance matrix of the VAR process. This leads to the definition of rPDC



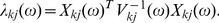
(7)


If 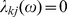, a Granger-causal linear influence from 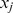 to 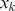 taking all other processes into account can be rejected at frequency 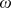. The 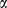-significance level for 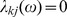 is given by 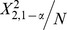 [17], which depends on the sample size and it is constant across frequencies. Based on this threshold the strength of interaction can be evaluated, e.g., for the purpose of comparing the strength at different frequencies or between pairs of variables.

### Preprocessing and rPDC analysis

In the off-line analysis, each of the original 19 common reference EEG channels (Fp1, Fp2, F7, F3, Fz, F4, F8, T3, C3, Cz, C4, T4, T5, P3, Pz, P4, T6, O1, O2) was re-referenced to a Hjorth type of reference, i.e., to the local average reference. In the case of a common reference montage the electrical activity at the reference can never be constantly zero and as a consequence it affects measurements at all other electrode sites. In addition, local average reference aims to accentuate local brain activity.

For Granger causality methods based on VAR modeling reliable estimation of model parameters in equation (1) is critical. In this study, we have carefully approached this part by examining both the stability of the model and the normality and uncorrelatedness of the residuals. Of importance is also the selection of an optimal model order that can capture the hidden connectivity patterns in a time-varying setup by avoiding over fitting. In practice, large model orders may lead to unstable situations or poor parameter estimates, while too small may not provide adequate description of the causal relationships. Computationally, this also depends on the number of analysis channels and on the available sample size. In order to obtain a reasonably small model for estimation and at the same time to capture dynamic changes of connectivity, the signals were first re-sampled (90 Hz) and then a sliding window approach was used (60 sec window, 5 sec steps). Each window was then zero-mean and variance normalized by dividing with the standard deviation [17], [23]. Down sampling was performed here to avoid unreliable parameter estimation, since with the original sampling frequency especially stability requirement of the model was not satisfied throughout the measurements.

**Figure 1:**
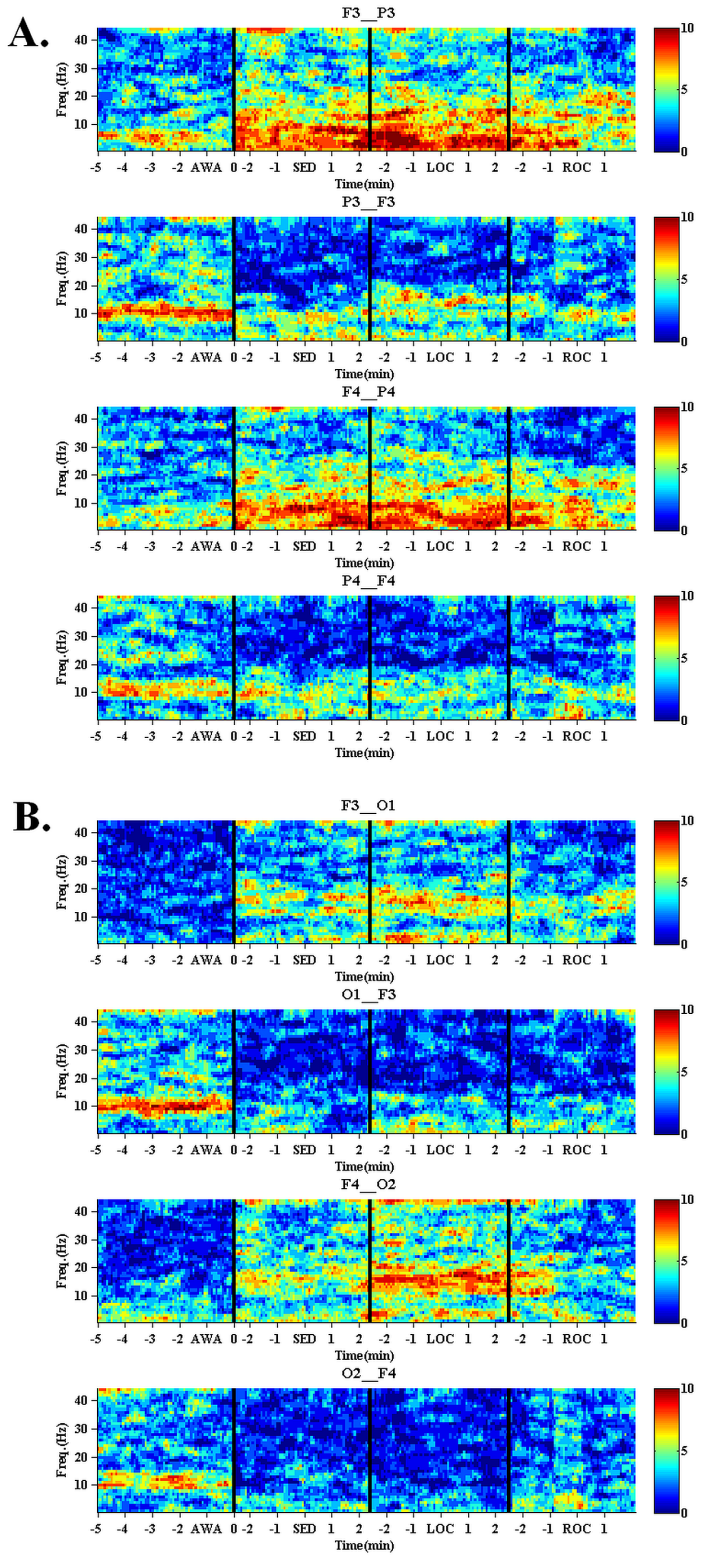
Directed connectivity between frontal and parietal brain regions. Fronto-parietal (F3–P3, F4–P4) (1a) and Fronto-occipital (F3–O1, F4–O2) (1b) directional connectivity during the awake (AWA) period compared to propofol-induced sedation (SED), propofol-induced loss of consciousness (LOC) and return of consciousness (ROC) after the termination of propofol infusion. The time frequency plot presents the number of subjects exceeding the significance level. Remark that the timeline between SED and LOC is almost continuous as all the subjects lost consciousness approximately 5 min after SED.

Identification of an optimal model order for estimation was based on subject-by-subject comparison of non-parametric and parametric estimates of the power spectra, examination of the fitting error and the statistical properties of the residuals. Stability of each model was always carefully examined. During this procedure one channel (Cz) was excluded from the final analysis. Finally, a model with 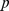 = 22 and the remaining 18 channels was selected for all subjects and EEG epochs. The connectivity criterion in the present analysis was the rPDC value exceeding the significance level (at 5% risk), which is 0.0011 (*N* = 5400). Results in the time frequency plots (Figure 1) are shown as the number of subjects exceeding this significance level.

### Statistical analysis

For statistical analysis, rPDC values were calculated from the following periods:

-Awake (AWA): 30 sec period starting 2 min before propofol infusion was initiated

-SED: 30 sec period ending 1 min before last testing when subject still responded

-LOC: 30 sec period ending 1 min before LOC-testing

-ROC: 30 sec period ending 1 min after ROC-testing

The time windows were chosen in order to exclude the potential interference of the LOC-testing (verbal command to open eyes) on the EEG signal. The rPDC values were then log-transformed to meet the assumption of normality prior to statistical analysis. rPDC values were analyzed with repeated measures analysis of variance (RM ANOVA) having the level of consciousness as a within-factor. A two-tailed p-value of less than 0.05 was considered statistically significant and to avoid multiplicity, Bonferroni correction was applied. Statistical analysis was conducted using SPSS, version 16.0 (SPSS Inc, Chicago, IL).

## Results

Drug administration was successful (i.e. LOC was reached) in all 10 subjects. No adverse events or clinically significant changes in the vital parameters were observed (data not shown).

**Figure 2:**
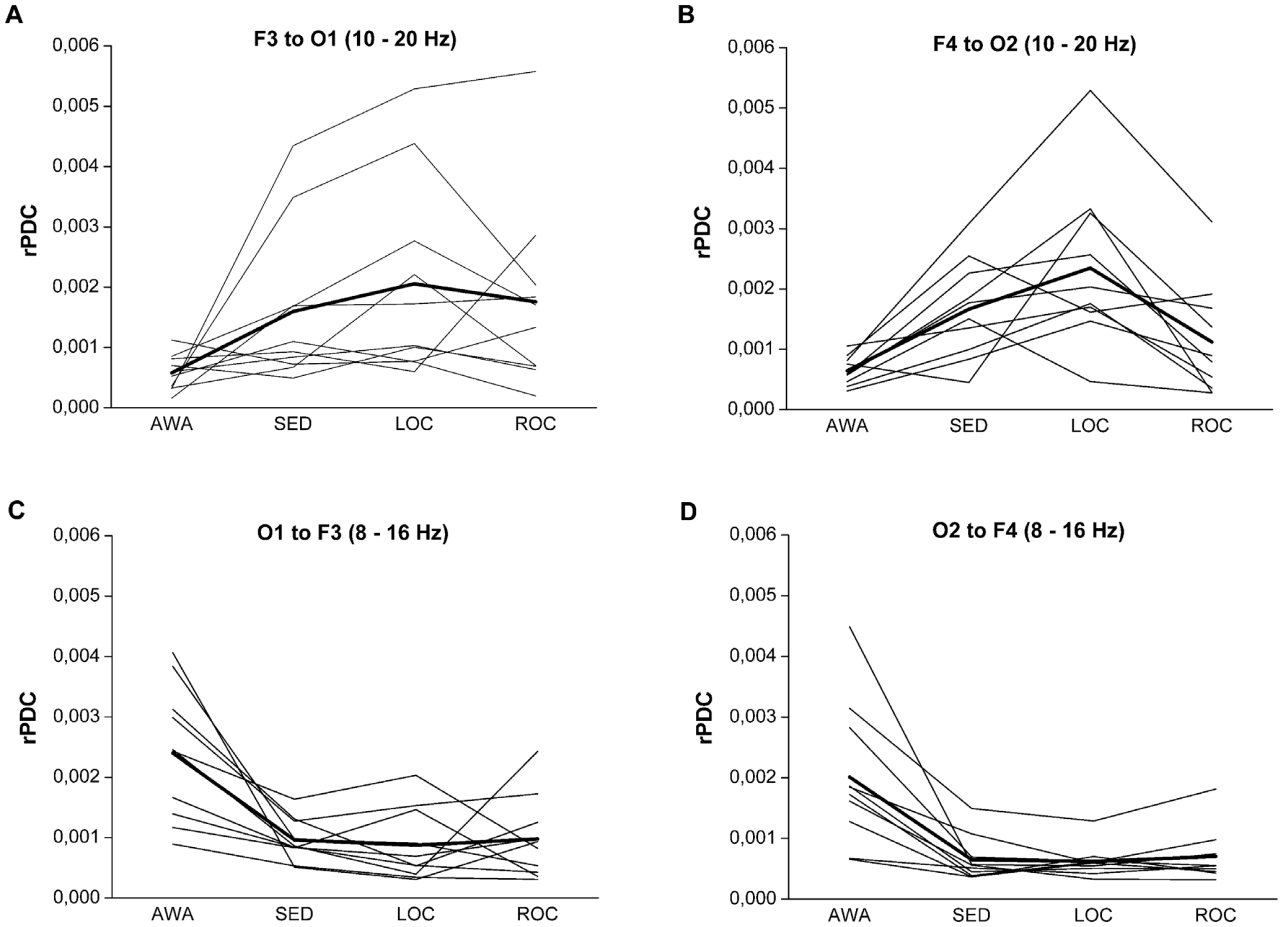
Individual and mean rPDC values at different stages of the study. Individual fronto-occipital (F3–O1, F4–O2) 10–20 Hz and occipito-frontal (O1–F3, O2–F4) 8–16 Hz rPDC values during stepwise increased propofol infusion during the awake (AWA) state, sedation (SED), loss of consciousness (LOC) and return of consciousness (ROC). Individual and mean rPDC values are presented with thin and thick lines, respectively.

From all the investigated channel pairs, the fronto-occipital (F3-O1, F4-O2) and fronto-parietal (F3-P3, F4-P4) pairs showed clearest propofol-induced changes in rPDC. These channel pairs were then chosen for further analyses. EEG frequency bands were selected based on visual inspection of the time frequency plots presenting the number of subjects exceeding the significance level (Figure 1) and rPDC values were computed accordingly. The results of the statistical analysis are presented in Table 1. Noticeable, a large dispersion of individual rPDC values was observed between the subjects as shown in Figure 2.

**Table 1:** Renormalized partial directed coherence (rPDC) values during propofol infusion.

	Band	rPDC AWA	rPDC SED	rPDC LOC	rPDC ROC	F	ANOVA	Paired comparisons		
	Hz	mean (std)	mean (std)	mean (std)	mean (std)			(Bonferroni-corrected)		
								AWA-SED	AWA-LOC	AWA-ROC
**F3**–**O1**	10–20	0.0006 (0.0003)	0.0016 (0.0013)	0.0021 (0.0016)	0.0018 (0.0016)	6.024	0.006	0.040	0.010	[0.132]
**F4**–**O2**	10–20	0.0006 (0.0002)	0.0017 (0.0008)	0.0023 (0.0013)	0.0011 (0.0009)	10.823	<0.001	0.006	0.001	[1.000]
										
**O1**–**F3**	8–16	0.0024 (0.0011)	0.0010 (0.0004)	0.0009 (0.0006)	0.0010 (0.0007)	8.577	0.001	0.003	0.004	0.012
**O2**–**F4**	8–16	0.0020 (0.0012)	0.0006 (0.0004)	0.0006 (0.0003)	0.0007 (0.0004)	8.485	0.001	0.002	0.002	0.005
										
**F3**–**P3**	1–12	0.0015 (0.0008)	0.0029 (0.0016)	0.0035 (0.0015)	0.0016 (0.0011)	7.693	0.002	[0.272]	0.003	[1.000]
**F4**–**P4**	1–12	0.0014 (0.0009)	0.0024 (0.0017)	0.0028 (0.0020)	0.0015 (0.0006)	2.763	[0.070]			
										
**P3**–**F3**	8–16	0.0022 (0.0010)	0.0014 (0.0008)	0.0017 (0.0009)	0.0013 (0.0008)	2.101	[0.139]			
**P4**–**F4**	8–16	0.0015 (0.0009)	0.0015 (0.0007)	0.0013 (0.0008)	0.0011 (0.0007)	0.692	[0.569]			

With overall significant (p<0.05) ANOVA result, the p-values for paired comparisons are also given. The non-significant p-values (p>0.05) are given in brackets. Abbreviations: ANOVA  =  Analysis of variance, AWA  =  Awake, SED  =  Sedation, LOC  =  Loss of consciousness, ROC  =  Return of consciousness.

In the back-to-front direction, propofol induced a bilateral decrease in the occipital-to-frontal 8 – 16 Hz rPDC (Table 1 and Fig. 2). After initial decrease from the AWA values during SED, mean rPDC remained low through LOC and ROC (Fig. 2, C–D). All conditions (SED, LOC, ROC) associated with significantly lower occipital-to-frontal rPDC than that measured during AWA (Table 1). However, no significant differences were detected between the SED, LOC and ROC states in the occipital-to-frontal rPDC.

In the front-to-back direction (frontal-to-occipital and frontal-to-parietal), the mean rPDC tended to follow the adjustments of propofol target concentration level (first gradually increasing during SED and LOC, followed by a decrease towards AWA-values during ROC). This phenomenon is depicted for the frontal-to-occipital channel pairs in Figure 2 (A-B). When compared to AWA, the increases in the frontal-to-occipital rPDC (10–20 Hz) reached statistical significance bilaterally at LOC but also during SED (Table 1). In the frontal-to-parietal direction, rPDC (1–12 Hz) increased significantly at LOC on the left side of the brain. No significant differences were detected between the LOC and SED states in the frontal-to-occipital and frontal-to-parietal rPDC.

## Discussion

In this study, rPDC was applied to assess how EEG directional connectivity is affected by slow induction of unconsciousness with anesthetic agent propofol. Despite the large dispersion of individual rPDC values, mean front-to-back rPDC expressed a concentration-dependent increase during propofol administration with a simultaneous decrease in rPDC in the opposite direction. Consequently, back-to-front connectivity dominance seen at awake state changed towards a more frontal-driven pattern during drug infusion. Importantly, no significant changes were detected between the SED and LOC states thus indicating that rPDC was not able to differentiate consciousness from unconsciousness.

A variety of methods have been proposed in literature for the study of directed connectivity in the brain. Some techniques rely on the specification of a model including structural parameters while others involve time series analysis techniques including causality measures [27]. A review of multivariate methods based on Granger causality can be found in [23], where the limitations of bivariate methods have also been described. In addition, the determination of causal relationships in the frequency domain is very important since brain rhythms have different role in information processing. In the study by Taxidis and coworkers (2010), cortico-hippocampal functional connectivity was assessed from local field potential measurements in the hippocampus and the medial prefrontal cortex in isoflurane-anesthetized rats using the generalized PDC approach [18]. Our study probably represents the first attempt in using multivariate causality methodology in anesthetized humans.

One recently published study using bivariate Granger causality observed an increase in front-to-back directed connectivity in anesthetized surgical patients [28]. However, this study applied a very liberal anesthetic regimen not confined to any particular agent; all 21 patients were given a rapid 2–4 mg/kg intravenous bolus dose of propofol during induction. For most patients, anesthesia was thereafter maintained with the infusions of propofol and remifentanil until the end of surgery. Two patients, however, received sevoflurane inhalation for anesthesia maintenance, and even nitrous oxide was used for some patients. Nevertheless, despite the obvious study design differences, congruence of the results with our study is evident. Further evidence for the usefulness of Granger causality based methods in EEG connectivity studies during anesthesia was recently presented by Barrett *et al.* (2012), where they detected increased bidirectional Granger causality and phase synchrony during propofol-induced anesthesia between two areas of interest, the anterior and posterior cingulate cortices [19]. Although other studies [9], [11] have reported diminished connectivity, the results of Barrett *et al.* support the main results of the present study.

In our study the main direction of information flow during normal conscious wakefulness was from posterior sensory and associate cortices to the frontal executive regions (back-to-front). Interestingly, the results of other human studies [9], [11] regarding this issue differ quite radically from our findings by showing front-to-back (frontal-to-parietal) information flow dominance during the awake state. However, in these studies on propofol-induced effects on directed connectivity, consciousness was tested every 5 s during the induction of anesthesia by requesting the subjects to open their eyes. Such a frequent task performance requires almost constant information processing and leads to highly increased executive workload in the brain. In our study, consciousness was tested every 5 min probably resulting in a more relaxed state of wakefulness and more steady and uncorrupted signal quality. Thus, the obtained opposing directions of information flow during the awake state could be explained by design differences of these studies.

Previous studies with contradictory results to the present findings have also used a more rapid induction of anesthesia [9], [11]. A 2 mg/kg intravenous bolus dose of propofol administered over a period of 20 s in Lee *et al.* resembles the normal clinical practice for anesthesia induction and results in LOC usually within one minute [9]. Although Ku *et al.* used a slightly slower induction with their TCI-protocol starting with a propofol target concentration of 2.0 µg/ml followed by 1.0 µg/ml increases every 20 s [11], the TCI-infusion scheme used in the present study clearly provided more gentle induction of anesthesia. Our approach was based on small stepwise dose increments at 10 min intervals resulting in subtle approach towards LOC through conscious sedation. With this infusion scheme we avoided dose overshooting at LOC, which could ultimately lead to varying states of brain suppression resulting in unequal LOC endpoints. Furthermore, our experimental setting allowed data acquisition during single-agent propofol anesthesia without any additional medication that could have had an effect on the EEG.

Methodological differences may also be responsible for the partly conflicting results. The EEG-reference problem, for instance, is inherent in all functional connectivity studies [29], [30], [31], [32]. Since changing the reference influences the correlation structure of the data set, some authors do not recommend the use of Laplacian type of reference, like the one used here, prior to multivariate autoregressive modeling [23]. Others, however, move even one step forward and perform similar connectivity analysis on cortical source reconstructed signals [33]. In addition, preprocessing such as filtering or re-sampling as well as noise, artifacts, and non stationarities in the signals can alter connectivity results [34], [35], [36].

In this study, we did not systematically examine all the above mentioned important issues. However, concerning the application of methods based on multivariate Granger causality it is noticeable, that during eyes closed wakefulness a back-to-front propagation has been found in several studies [37], [22], [38], similar to our results. In these studies a common reference was used and different preprocessing and model orders were applied. In Babiloni *et al.* (2008) it was further discussed that the preponderant parietal-to-frontal over frontal-to-parietal EEG functional coupling may reflect a background flux of sensory signals from parietal to frontal areas [38]. Furthermore, in Kus *et al.* (2004) it was pointed out that with bivariate measures this pattern can be disorganized and even the reversal of propagation may be observed [22]. In addition, in Kaminski *et al.* (1997) it was shown that during sleep (sleep level 3 and 4) EEG activity spreads from the fronto-central region and the authors have suggested, that it may be due to the influence of sub-cortical regions [37]. Finally, in a recent study of Purdon *et al.* (2013) loss of consciousness was marked by the loss of spatially coherent occipital alpha oscillations and the appearance of spatially coherent frontal alpha oscillations [39]. In this study a combined spectral and global coherence analysis was performed with local average reference [27]. Apparently, these regions correlate with the “source” areas of propagation found in our study.

The observed decrease of connectivity in the back-to-front direction (8–16 Hz) is of interest considering the current understanding of how anesthesia induces impairment of perception. Failure of conscious understanding of the surrounding world is thought to be a result of decreased cortical connectivity leading to a state where information is received but not perceived [3], [40]. The observation of decreased information flow from the primary sensory and associative cortices of the posterior brain to more anterior executive areas could be interpreted to support this view of perception failure. Decreased cortical connectivity limits our ability for rich contents of consciousness because the executive regions no longer have uninterrupted access to processed information from the environment. This is also supported by a recent study where information processing capacity in the posterior brain regions was significantly disturbed after induction of anesthesia with propofol, whereas the frontal network was affected only minimally [10]. Moreover, our results support the idea that some sensory processing is present even after LOC [40] as well as the earlier suggestion that primitive consciousness is more relied on the deep, phylogenetically old, brain structures while the cortex contributes to the rich contents of consciousness [8], [41].

While the decrease in back-to-front mean rPDC remained at sedative levels throughout propofol administration, the increase in the opposite (front-to-back) direction seemed to follow the target concentration of propofol infusion. This, and the fact that no significant rPDC differences were observed between the sedative- and the LOC-states, suggests that at least the observed connectivity increases were in fact due to the drug and not due to changes in the level of consciousness. The observed rPDC changes in the frontal-to-occipital direction seem to inversely follow the well-known suppression pattern seen in the brain metabolism and blood perfusion during propofol anesthesia [8], [42]. This brings forth an important issue commonly overlooked in consciousness studies utilizing anesthetic drugs: anesthetics possess brain effects distinct from the effect related to their ability to induce unconsciousness. Functional connectivity between the frontal and parietal regions has been suggested to be important for conscious perception [3], [9], [43]. Thus, it is somewhat surprising that we did not see any reduction in cortical connectivity within frontoparietal network at LOC compared to ROC or SED.

There are a few limitations in our study, which have to be acknowledged. We studied only one drug (propofol) in a rather small group of healthy male subjects and possible differences between anesthetic agents and different demographic groups remain to be studied. Our study can also be criticized for not studying higher, surgical doses of propofol. Our maximum concentration level was targeted to LOC defined as loss of motor response to a verbal command. Unresponsiveness does not necessarily mean unconsciousness [44], and studying supramaximal doses could have shed light to this philosophical problem. There are, however, ethical constraints to induce deeper levels of anesthesia in the absence of medical indication. Also, burst-suppression pattern in EEG induces more complexity for any connectivity analysis as the frequency content of EEG is intermittently alternating at this deep level of anesthesia. It should be emphasized, however, that none of the ten subjects reported of being conscious during unresponsiveness when interviewed several times after the study session.

In this study we utilized rPDC analyses for the study of causal relationships in EEG during gradual and slow progression to unconsciousness in humans. Our results indicate that rPDC analysis of the EEG signal can detect anesthetic-induced changes in directional interactions and causal links within cortical networks. However, cortical directed connectivity did not seem to be able to differentiate consciousness from unconsciousness, putting the previous claims for neural correlates of anesthetic-induced unconsciousness on hold. As for the creation of better depth-of-anesthesia monitoring devices in the future, the ultimate question remains: to what extent (if any) do the observed EEG signal alterations reflect the changes in consciousness?
